# A Variable Polyglutamine Repeat Affects Subcellular Localization and Regulatory Activity of a *Populus* ANGUSTIFOLIA Protein

**DOI:** 10.1534/g3.118.200188

**Published:** 2018-06-08

**Authors:** Anthony C. Bryan, Jin Zhang, Jianjun Guo, Priya Ranjan, Vasanth Singan, Kerrie Barry, Jeremy Schmutz, Deborah Weighill, Daniel Jacobson, Sara Jawdy, Gerald A. Tuskan, Jin-Gui Chen, Wellington Muchero

**Affiliations:** * Biosciences Division and BioEnergy Science Center, Oak Ridge National Laboratory, Oak Ridge, TN 37831; † Center for Bioenergy Innovation, Oak Ridge National Laboratory, Oak Ridge, TN 37831; ‡ U.S. Department of Energy Joint Genome Institute, Walnut Creek, CA 94598; § HudsonAlpha Institute for Biotechnology, Huntsville, AL 35806; ** The Bredesen Center for Interdisciplinary Research and Graduate Education, University of Tennessee, Knoxville, Knoxville, TN 37996

**Keywords:** PolyQ, subcellular localization, cell wall, lignin, *Populus*

## Abstract

Polyglutamine (polyQ) stretches have been reported to occur in proteins across many organisms including animals, fungi and plants. Expansion of these repeats has attracted much attention due their associations with numerous human diseases including Huntington’s and other neurological maladies. This suggests that the relative length of polyQ stretches is an important modulator of their function. Here, we report the identification of a *Populus* C-terminus binding protein (CtBP) ANGUSTIFOLIA (*PtAN1*) which contains a polyQ stretch whose functional relevance had not been established. Analysis of 917 resequenced *Populus trichocarpa* genotypes revealed three allelic variants at this locus encoding 11-, 13- and 15-glutamine residues. Transient expression assays using *Populus* leaf mesophyll protoplasts revealed that the 11Q variant exhibited strong nuclear localization whereas the 15Q variant was only found in the cytosol, with the 13Q variant exhibiting localization in both subcellular compartments. We assessed functional implications by evaluating expression changes of putative *PtAN1* targets in response to overexpression of the three allelic variants and observed allele-specific differences in expression levels of putative targets. Our results provide evidence that variation in polyQ length modulates *Pt*AN1 function by altering subcellular localization.

The link between variable trinucleotide repeat expansion and changes in protein function has been reported across diverse organisms including humans, fungi and plants. For example, onset and progression of numerous human diseases exhibit high correlation with the presence of trinucleotide repeats (Butland *et al.* 2007; La Spada and Taylor 2010). Among these, polyglutamine (polyQ) repeats, encoded by the trinucleotides CAG, have been implicated in eleven different human diseases (La Spada and Taylor 2010). In most cases, it has been shown that these long polyQ-harboring proteins form aggregates within the nucleus and this aggregation leads to protein dysfunction, or in some cases gain of function leading to disease onset (Orr 2012; Karlin and Burge 1996; Gatchel and Zoghbi 2005; Buchanan *et al.* 2004). Based on their over-representation in transcription factors across diverse organisms, it has been proposed that polyQ repeats are under strong selective pressure (Gerber *et al.* 1994; Willadsen *et al.* 2013; Whan *et al.* 2010), suggesting that these features underlie critical functions in proteins.

In a study of a chimeric *GAL4* transcription factor, Gerber *et al.* (1994) reported a positive correlation between length of a polyQ tract with *GAL4* transcriptional activity when expressing a series of GAL4 chimeras containing progressively longer stretches of glutamines in HeLa cells. Additionally, there is evidence suggesting that polyQ tracts may function in protein-protein interactions (Schaefer *et al.* 2012). In this regard, it has been proposed that expanded repeats stabilize coil-coiled protein interaction domains and that the length of the tract can impact binding properties of the protein (Schaefer *et al.* 2012; Willadsen *et al.* 2013). Beyond transcriptional regulation, polyQ repeats themselves have been linked to phenotypic trait variation. In an analysis of spawn timing in salmon, it was shown that variation in the length of a repeat within a clock gene was correlated with variation in spawn timing (O’Malley and Banks 2008). Similarly, length polymorphism of the same repeat in a circadian clock gene was also associated with fecundity and variation in timing of breeding in avian species (Caprioli *et al.* 2012). In this case, a single extra glutamine in the repeat region led to later breeding times in female birds heterozygous for the longer allele. Not only does this provide evidence for the effect of a single amino acid difference in repeat length on a trait, but also that allelic variants can act in a dominant manner, which has been postulated to occur in human diseases as well (La Spada *et al.* 1991; MacDonald *et al.* 1993; Orr *et al.* 1993).

The presence of polyQ repeats and modulation of phenotypic expression has also been reported in plants (Kottenhagen *et al.* 2012; Rival *et al.* 2014; Undurraga *et al.* 2012). Although polyQ repeats in plants, on average, are not as long as those found in animal genomes, there have been several reports for selective pressures acting on these repeats leading to obvious functional changes. For example, *PHYTOCHROME AND FLOWERING TIME 1* (*PFT1*) in *Arabidopsis* possesses highly conserved short tandem polyQ repeats that appear to be under constrained selection to maintain proper protein function (Rival *et al.* 2014). Deletion of this feature resulted in transgenic plants expressing a similar flowering phenotype with loss-of-function mutants in *Arabidopsis* (14). In another example, polyQ repeats were shown to be highly variable within the protein *EARLY FLOWERING 3* (*ELF3*) across *Arabidopsi*s species (Undurraga *et al.* 2012). It was further demonstrated that polyQs of different lengths had variable success in rescuing a particular *Arabidopsis* accession with a loss-of-function *efl3* background, indicating that the genotypic background had a prominent effect on *ELF3* function. In tree species, a polyQ repeat in a *CONSTANS*-like (*COL*) gene is involved in phenology and growth in North American red oak (Lind-Riehl *et al.* 2014). In *Populus tremula*, one allele of a polyQ repeat in the *COL2B* gene is associated with growth cessation (Ma *et al.* 2010). These cumulative observations suggest that polyQ repeats evolved to modulate protein function in diverse molecular processes. A consistent theme in these studies has been that variation in polyQ length can have major implications for protein function.

In this study, we sought to determine the functional consequences of polyQ repeat variation found in the *Populus C-TERMINAL BINDING PROTEIN* (*CtBP*) *ANGUSTIFOLIA* (*AN*)-encoding gene, Potri.014G089400, hence forth referred to as *PtAN1*. This repeat exhibited length polymorphism in a natural population of *Populus trichocarpa* genotypes with three predominant allelic variants encoding 11-, 13-, 15Q repeats. Variants at this locus exhibited significant association with 6-carbon sugar content, xylose and glucose release across multiple environments in a previous genome-wide association mapping study (GWAS) (Muchero *et al.* 2015). In that study, transient overexpression in leaf mesophyll protoplasts revealed that allelic variants differed in their ability to induce expression of cell wall biosynthesis marker genes *CCoAOMT1* and *CesA8*. Based on these observations, we sought to establish the mechanism behind the apparent differences in transcriptional regulation.

## Materials and Methods

### Sequence analysis

*Populus trichocarpa* natural variant association mapping population has been described previously (Muchero *et al.* 2015). Analysis of polyQ variation in *Pt*AN1 (Potri.014G089400) is based on resequencing of this population. Paralogs of *Pt*AN1 in other plant species were determined through BLAST alignments from Phytozome database v10.3 (phytozome.ji.doe.gov/pz/portal.html). Non-plant CtBP sequences were obtained from NCBI (www.ncbi.nlm.nih.gov) and included HsCtBP (AAC62822), MmCtBP (NP_001185788), XlCtBP (NP_001079151) and DmCtBP (BAA25287). A phylogenetic tree was created in MEGA software (Tamura *et al.* 2011) using the Maximum-Likelihood method and Bootstrap values were calculated from 1000 independent runs.

### Plant materials

*Arabidopsis* plant materials were obtained from the *Arabidopsis* Biological Resource Center (ABRC). The *Arabidopsis* Columbia (Col-0) ecotype was utilized as control and the *ANGUSTIFOLIA* T-DNA mutant line *an-t1* (TAIR stock CS851381) was described previously (Gachomo *et al.* 2013). Genotyping of the *an-t1* lines was carried out utilizing 3-primer Polymerase Chain Reactions methods utilizing NEB taq (New England Biological). Primers for genotyping *an-t1* lines were an-t1F 5′ GAATGTCGGTAACGTAGTGGGT, an-t1R 5′ ACTTTCTCCCTGTTGCCTACTG, and p745 5′ AACGTCCGCAATGTGTTATTAAGTTGTC.

### Co-evolution analysis

The correlation between the occurrence of all pairs of SNPs in the ANGUSTUFOLIA paralogs as well as SNPs found elsewhere in the genome across 917 *P. trichocarpa* genotypes was calculated using the CCC correlation metric (Joubert *et al.* 2017; Climer *et al.* 2014), an allele-specific SNP correlation metric. An MPI-wrapper was written around the CCC software (Climer *et al.* 2014) in order to parallelize it for use on the Oak Ridge Leadership Computing Facility clusters, making use of the Parallel::MPI::Simple Perl module, developed by Alex Gough and available on The Comprehensive Perl Archive Network (CPAN) at http://search.cpan.org/∼ajgough/Parallel-MPI-Simple-0.03/Simple.pm. The application of a threshold of 0.7 resulted in a network (referred to as the SNP co-evolution network) in which each node represented a SNP and each edge represented the correlation between two SNPs, potentially indicating a co-evolution relationship. SNPs were mapped to the genes in which they were present resulting in a gene co-evolution network in which two genes were considered to be potentially co-evolving if the one gene contained a SNP that was correlated with a SNP in the other gene. Connected components of the resulting co-evolution network which included an ANGUSTUFOLIA paralog were extracted using the Perl Graph module available from http://search.cpan.org/dist/Graph/lib/Graph.pod. Networks were visualized in Cytoscape (Shannon *et al.* 2003).

### Co-expression analysis

Gene expression (FPKM) values for each of the tissues types and perturbations contained in the *P. trichocarpa* Gene Atlas were obtained from Phytozome (Goodstein *et al.* 2012) and used to create an expression vector for each gene. The Pearson correlation coefficient (PCC) was calculated for all pairs of genes using the mcxarray and mcxdump programs from the MCL-edge package (Van Dongen 2008) which can be obtained from http://micans.org/mcl/. Thresholds of 0.9 and 0.95 were applied. The resulting correlations were used as edge weights to form a *P. trichocarpa* co-expression network.

### Arabidopsis RNA-Seq profiling

Stranded RNA-Seq libraries were generated and quantified using qPCR. Sequencing was performed on an Illumina HiSeq 2500 (150 bp paired end sequencing). Raw fastq file reads were filtered and trimmed using the JGI QC pipeline. Using BBDuk (https://sourceforge.net/projects/bbmap/), raw reads were evaluated for sequence artifacts by kmer matching (kmer = 25) allowing 1 mismatch and detected artifacts were trimmed from the 3′ end of the reads. RNA spike-in reads, PhiX reads and reads containing any Ns were removed. Quality trimming was performed using the phred trimming method set at Q6. Following trimming, reads under the length threshold were removed (minimum length 25 bases or 1/3 of the original read length; whichever was longer). Raw reads from each library were aligned to the *Arabidopsis* reference genome using TopHat2 (Kim *et al.* 2013). Only reads that mapped uniquely to one locus were counted. FeatureCounts (Liao *et al.* 2013) was used to generate raw gene counts. Raw gene counts were used to evaluate the level of correlation between biological replicates, using Pearson’s correlation to identify which replicates would be used in the differential gene expression (DGE) analysis. DESeq2 (v1.2.10) (Love *et al.* 2014) was subsequently used to determine which genes were differentially expressed between pairs of conditions. The parameters used to “call a gene” between conditions were determined at a *p*-value <0.05. Functional classification of DEGs was performed using MapMan (Thimm *et al.* 2004) and Gene Ontology (GO). GO enrichment was performed using agriGO (Tian *et al.* 2017).

### Protoplast transfection and subcellular localization

To confirm the subcellular localization and transiently overexpress the *Pt*AN1 variant in the *Populus* cell, sequences for the variant Potri.014G089400 coding sequences were cloned from specific natural variants of *P. trichocarpa* genotypes. The 11Q variant was derived from BESC-20, 13Q variant from GW-9799 and 15Q variant from BESC-191 plant materials. RNA was isolated from leaf material from plants grown under greenhouse conditions. 100 mg of tissue was used for extracting RNA and with the above-mentioned protocol. cDNA was generated from 1 µg of RNA using Thermo Fisher Scientific first strand cDNA synthesis kit according to manufacture’s instructions. Potri.014G089400 coding sequences were cloned from resulting cDNA libraries using PHUSION polymerase (TAKARA) and cloned into pENTER D/TOPO (Invitrogen). Plasmids carrying variant sequences were then cloned into the pSATA6-DEST-YFP plasmid (CD1652 from ABRC) and used for protoplast transfection. Primers used for cloning were: PtAN1F 5′-CACCATGAGCGCCACGACTACCAGAT-3′, PtAN1R 5′-ATCTAGCCAACGAGTAACACCATC-3′.

Protoplast isolation and transfection was described previously (Guo *et al.* 2012). Briefly, we utilized *P. tremula* × *P. alba* clone ‘717-1B4’ grown in magenta box containers with MS medium. Leaves were collected, and protoplasts isolated as previously described. Approximately 1 × 10^4^ cells were co-transfected with YFP fused variant Potri.014G089400 sequences (11Q, 13Q and 15Q, respectively) and VirD2NLS-mCherry (nuclear marker) using the PEG method and incubated for 12-14 h in low light. For subcellular localization assay, imaging was carried out utilizing Zeiss 710 Meta Confocal and images taken using Zeiss ZEN software (Carl Zeiss).

### RNA extraction and qRT-PCR

RNA extraction from protoplasts was independently performed from three replicated transfections and isolated using a Spectrum Plant Total RNA isolation kit (Sigma) according to the protocol provided. The optional on-column DNase treatment was included during RNA isolation to rid the samples of potential genomic DNA contamination. Total RNA quantity and quality was determined using a NanoDrop spectrophotometer (Thermo Scientific). cDNA synthesis was carried out using a SuperScript III First-Strand Synthesis SuperMix for qRT-PCR (Invitrogen) according to the protocol provided. The resulting 20 μl of cDNA was diluted in 100 μl H_2_O and used for qRT-PCR.

qRT-PCR was performed using the StepOnePlus Real-Time PCR system (Applied Biosystems) with SYBER green reaction mix (Bio-Rad Life Sciences) according to manufactures recommendations for 20 µl reactions. Gene expression was calculated using the ΔΔCt method (Livak and Schmittgen 2001) with *UBIQUITIN 10b* for template normalization. Primers used in this study were listed in Table S2.

### Statistical Analysis

The statistical significance of differences in measured parameters was tested by using the procedures of DPS (Zhejiang University, China). Differences were compared using Duncan test and Fisher’s protected least significant difference (LSD) test at 0.05 probability levels.

### Data availability

The RNA-Seq sequencing data have been deposited in the NCBI Sequence Read Archive (SRA) under the accession number SRP123401. The authors state that all data necessary for confirming the conclusions presented in the article are represented fully within the article. Supplemental material available at Figshare: https://doi.org/10.25387/g3.6391991.

## Results

### Whole genome duplication and divergence of PtAN paralogs

**Figure 1:**
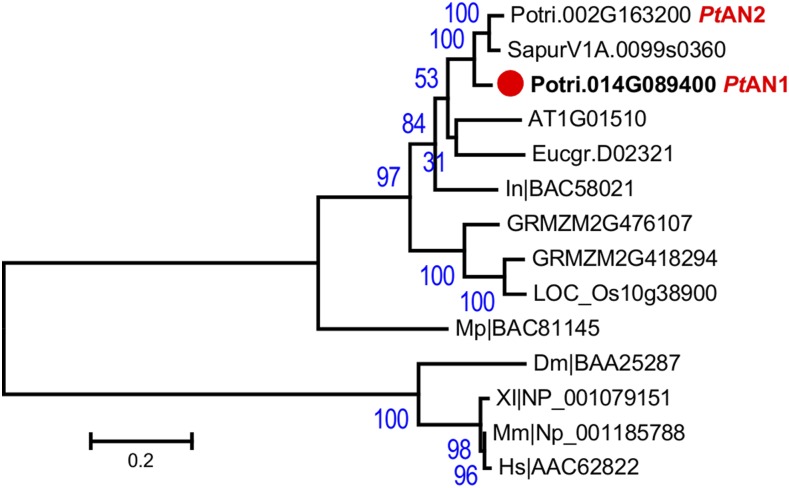
Phylogenetic analysis of *ANGUSTIFOLIA* gene family. *Populus trichocarpa* locus Potri.014G089400 shows the highest homology to the plant gene *ANGUSTOFOLIA* (*AN*) identified in *Arabidopsi*s (AT1G01510). *ANGUSTIFOLIA* in plants is a single copy gene and shows the highest homology to the animal CtBP/BARs gene. *Populus* has two *ANGUSTIFOLIA* paralogs, *Pt*AN1 (Potri.014G089400) and *Pt*AN2 (Potri.002G163200). Plants show a distinct relationship compared to animal CtBP. Bootstrap values are provided at branches.

Phylogenic analysis in the *Populus* reference genome assembly showed that *Pt*AN1 (Potri.014G089400) shared high homology with its paralog, *Pt*AN2 (Potri.002G163200) (Figure 1), which resulted from the salicoid genome duplication and rearrangement event (Tuskan *et al.* 2006). As reported previously, *ANGUSTIFOLIA/CtBP/BARS* genes exhibit extremely low levels of internal duplication typically occurring as single-copies in genomes of diverse organisms including plants and animals (Figure 1) (Kim *et al.* 2002). Sequence alignments of *PtAN1* and *PtAN2* compared to *AN/CtBP* homologs, revealed that, unlike other organisms, *PtAN1* and *PtAN2* encode proteins carrying polyQ repeats in their N-terminus region (Figure S1). Specifically, *PtAN1* carried 11 glutamine residues while *PtAN2* carried 2 residues in the *Populus trichocarpa* reference genome V3.1 (Figure S1).

### Natural variation of a PolyQ repeat in PtAN1

Since the longer polyQ repeat in *PtAN1* has only been observed in *Populus* thus far, we sought to establish natural variation of this unique feature by evaluating 917 resequenced *P. trichocarpa* genomes representing the range-wide distribution of the species in the Pacific Northwest region of North America (Evans *et al.* 2014).

**Figure 2:**
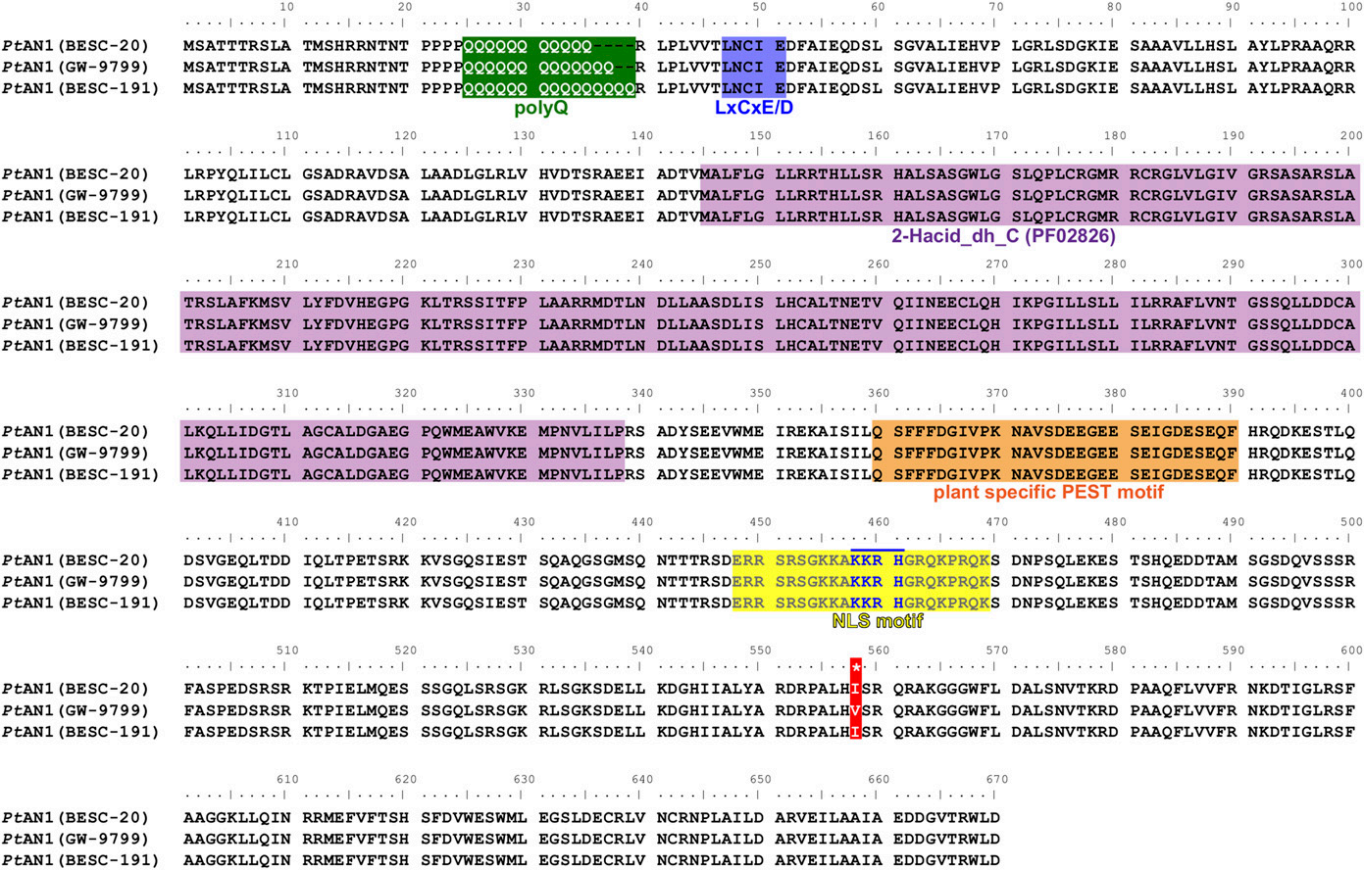
Sequence alignment for three alleles containing different variant size polyQ repeats of *Pt*AN1 from a population. Green background designates the polyQ region. Blue highlight shows a putative retinoblastoma binding site. Purple background represents conserved 2-HACID domain and orange highlight denotes a plant specific PEST domain. Amino acid sequence alignment shows the three sequences vary in the polyQ repeat region. A single amino acid difference observed between the 13Q and 11Q or 15Q alleles is denoted by *.

**Figure 3:**
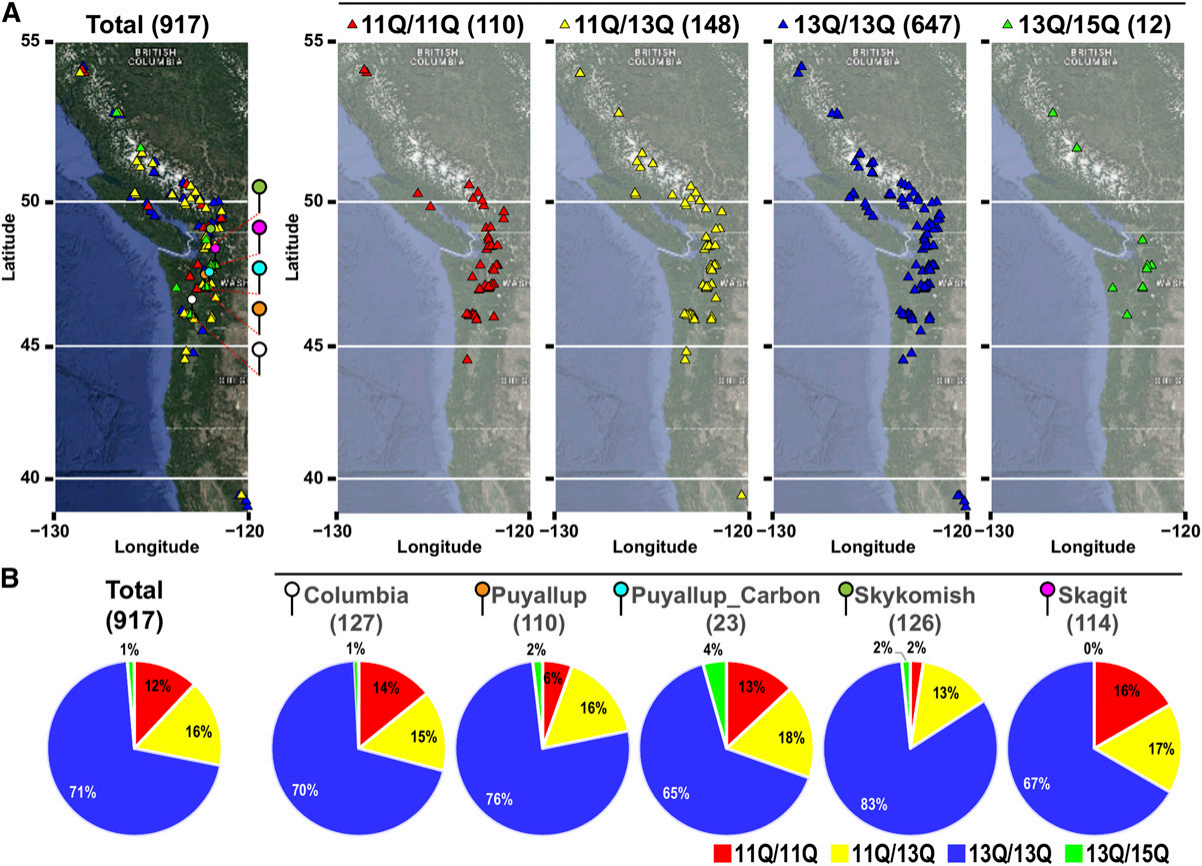
Frequency of allelic variants identified in population samples of *P. trichocarpa*. A) Geographic distribution of the genotypes. B) The frequency of three variants found in the populations. Genotype of homozygotes for 13Q variants is found with the highest frequency. No genotype carried homozygous 15Q alleles.

Significant variation in the length of this polyQ motif was observed with three alleles carrying 11-, 13- and 15Q repeats (Figure 2 and 3). Variants harboring the 15Q allele were only found as heterozygotes in combination with the 13Q allele in 12 genotypes while the second-most predominant 11Q variant was found in 110 and 148 homozygous and heterozygous individuals, respectively (Figure 3). The predominant allele, 13Q, was found in homozygous state in 647 and heterozygous in 148 individuals (Figure 3). Based on the geographic distribution of the alleles across the species range, the individual alleles appear to be uniformly distributed (Figure 3B). Since Illumina short read sequencing has been shown to be susceptible to high error rates in polyQ genotyping (Reumers *et al.* 2012), cDNAs for 11Q, 13Q and 15Q alleles were cloned and Sanger sequenced to eliminate the possibility of erroneous residue counts in downstream validation experiments. Alignment of these sequences confirmed the predicted variation in the polyQ region. Additionally, the 11Q and 15Q alleles were identical outside of this region whereas the 13Q allele had an additional single non-synonymous mutation (I to V 546 aa) (Figure 2).

### Subcellular localization of *Pt*AN1 is impacted by polyQ repeat length

**Figure 4:**
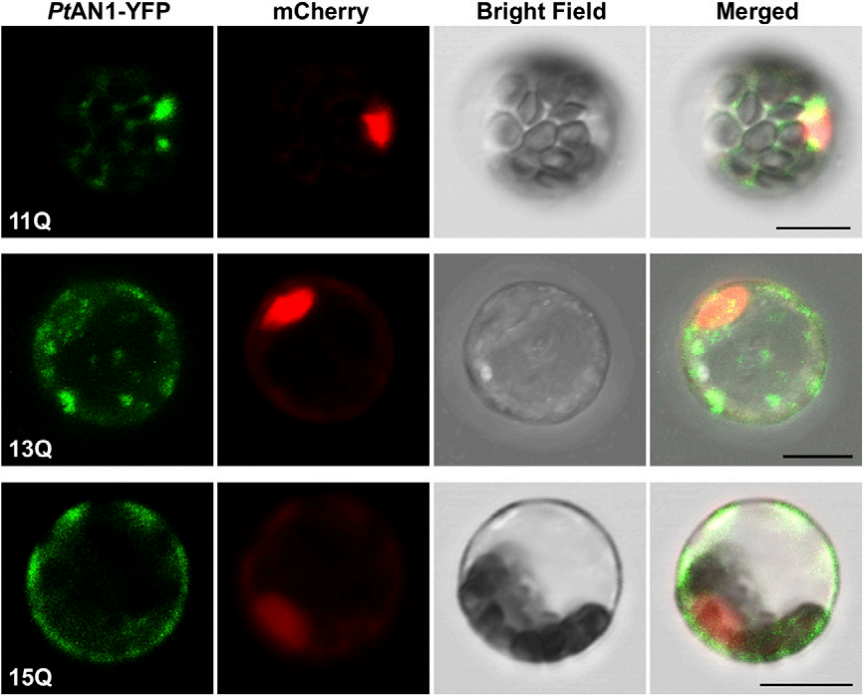
Subcellular localization of *Pt*AN1 in *Populus* protoplasts. YFP was fused with different alleles of *Pt*AN1. *35S:PtAN1-YFP* co-transfected with VirD2NLS-mCherry tagged nucleus marker into poplar mesophyll protoplasts. YFP signal is indicated as green color and mCherry signal is indicated as red color. Scale bar is 10 μm.

To assess the molecular basis of polyQ length modulating *Pt*AN1 function, we determined the subcellular localization of the three allelic variants, specifically focusing on the ability to localize the variants in the nucleus as supporting evidence for a putative role in transcriptional regulation. To do this, we utilized the *Populus* protoplast assay (Guo *et al.* 2012) and imaged localization of the YFP-fused proteins to determine subcellular localization of the *Pt*AN1 variants. The 11Q variant showed strong localization in the nucleus as well as some punctate localization in the cytoplasm (Figure 4). Interestingly, the 15Q variant showed no nuclear localization, but rather was restricted to punctate regions in the cytoplasm. On the other hand, the 13Q allele exhibited variable subcellular localization representing both cytoplasmic and nuclear localization (Figure 4). Based on these results, the difference in length of the polyQ repeat region had a strong impact on the ability of *Pt*AN1 to move into the nucleus in *Populus* protoplasts. These results support our previous observations that the 11Q and 13Q variants had significantly different activity in modulating the induction of *CesA8* and *CCoAOMT1* when overexpressed in *Populus* protoplasts (16). Since regulatory targets for *PtAN1* are largely unknown in *Populus*, we sought to use a combination of co-expression networks and RNA-Seq analyses on the *Arabidopsis AN* T-DNA null allele mutant (*an-t1*) as tools to infer putative targets.

### Expression regulatory networks of ANGUSTIFOLIA

**Figure 5:**
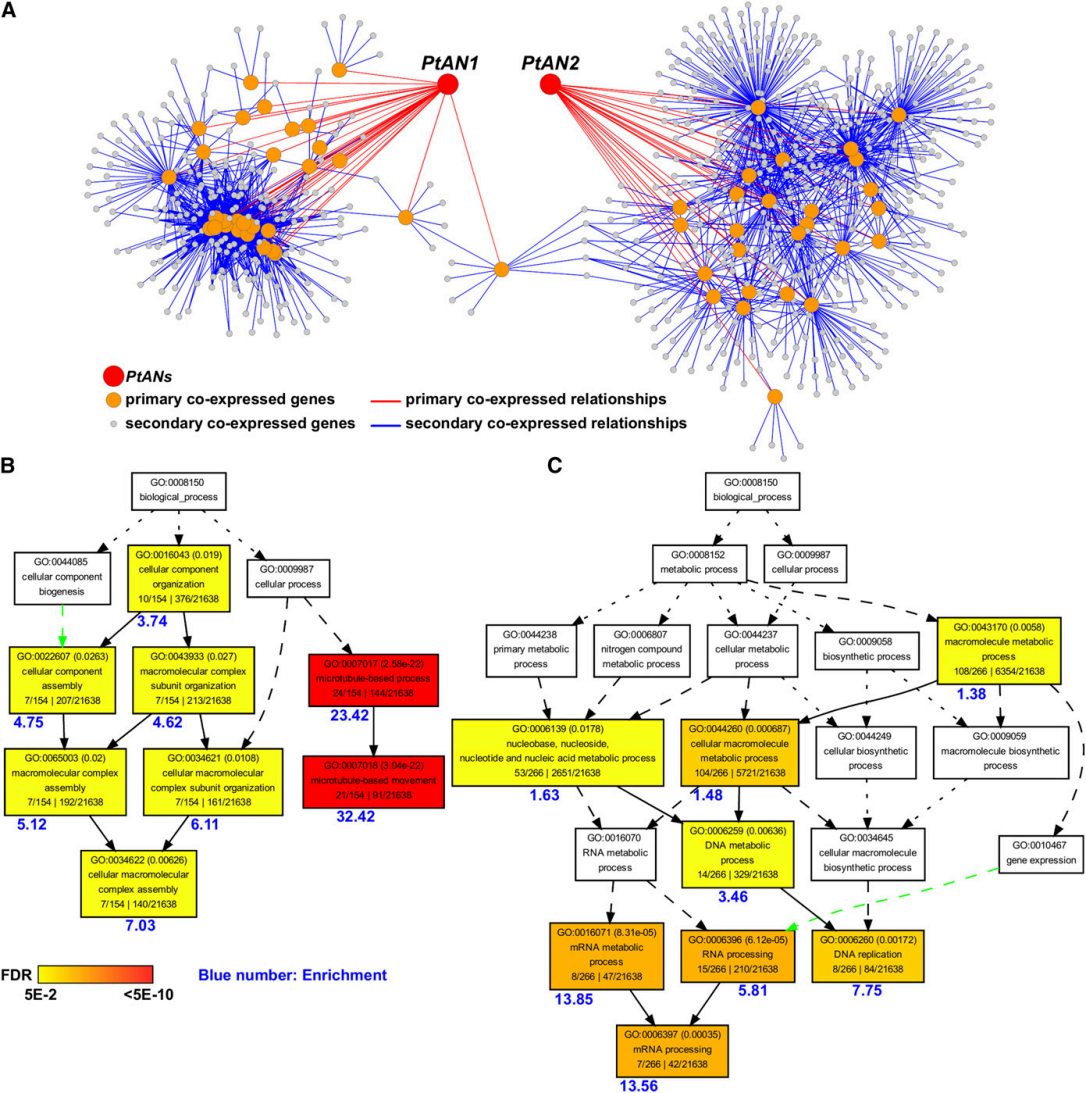
Co-expression network of *PtANs*. A) Co-expression network of *PtAN1* and *PtAN2*. B-C) Enriched GO terms of *PtAN1* (B) and *PtAN2* (C) co-expression networks.

**Figure 6:**
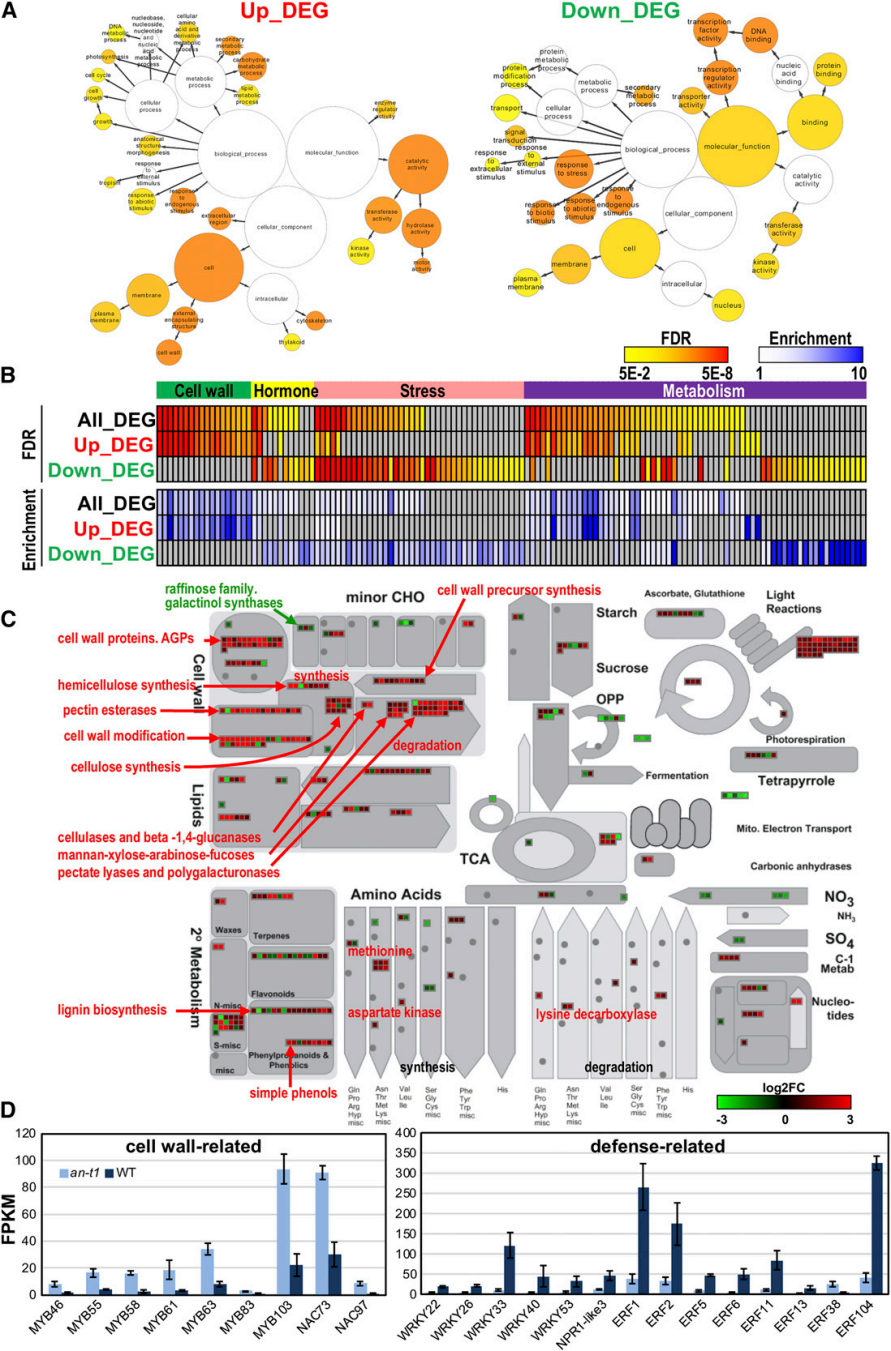
Functional classification of DEGs in *Arabidopsis an-t1* mutant. A) Enriched GO terms of up- or down-regulated DEGs in *an-t1* mutant. B) Functional classification of significant GO terms of DEGs. C) Expression patterns of DEGs involved in metabolism through MapMan. D) Expression of marker genes involved in cell wall and defense in *an-t1* mutant.

Co-evolution and co-expression analysis did not reveal any shared networks between *PtAN1* and *PtAN2*, suggesting that these loci may have undergone functional divergence (Figure S2). The co-expression network for *PtAN1* exhibited significant enrichment of microtubule-related processes and microtubule-related movement (Figure 5) and was consistent with observations in *Arabidopsis* where *AtAN* was shown to regulate the arrangement of cortical microtubules in leaf cells (Kim *et al.* 2002). Based on these results, *PtAN1* appears to be co-expressed with similar gene families previously described for *Arabidopsis AN*. To expand on this observation, we performed RNA-Seq analysis on the *Arabidopsis AtAN* T-DNA null allele mutant line (*an-t1*) and the Col-0 wild type. Differential expression analysis revealed significant up-regulation of genes involved in cell wall formation in the *an-t1* mutant (Figure 6). These included *MYB46*, one of the master regulators in cell wall biosynthesis (Zhong *et al.* 2007). On other hand, genes involved in defense signaling were significantly down-regulated. These included well characterized defense response transcription regulators such as *WRKY33* and multiple ethylene response factors (*ERFs*) (Figure 6D) (Gutterson and Reuber 2004; Zheng *et al.* 2006).

### PolyQ repeat length affects expression levels of putative targets in Populus protoplasts

To evaluate the molecular function of *PtAN1*, we first determined the relative gene expression profile using tissue-specific cDNA libraries isolated from poplar. We determined that *PtAN1* was expressed in all tissues with slightly higher expression in vascular tissues, root tip and female catkins (Figure S3). Based on *Arabidopsis* eFP browser (Winter *et al.* 2007), the *Arabidopsis AN* homolog is ubiquitously expressed throughout the plant, though visible phenotypes for *an-t1* mutants have only been described in leaf tissues.

Since *AN* is thought to act as a co-repressor in *Arabidopsis* (Kim *et al.* 2002; Gachomo *et al.* 2013), we evaluated the impact of alternate polyQ alleles on expression of putative target genes selected based on *Populus* co-expression and *Arabidopsis* RNA-Seq data. Six putative targets were selected based on implication in microtubule related processes and movement. In addition, their orthologs were also differentially expressed in the *Arabidopsis* RNA-Seq analysis. These included genes encoding a D-glucose binding protein (Potri.011G140000), P-loop containing proteins (Potri.010G069400 and Potri.013G020700), TUB8 protein (Potri.009G040200), TUA3 protein (Potri.001G004600), and an ATP binding microtubule motor protein (Potri.001G182300). From the *Arabidopsis* RNA-Seq, we selected *MYB46* (Potri.001G258700), *MYB83* (Potri.001G267300) and *WRKY33* (Potri.016G128300) since they represent key regulators of cell wall formation and defense signaling.

**Figure 7:**
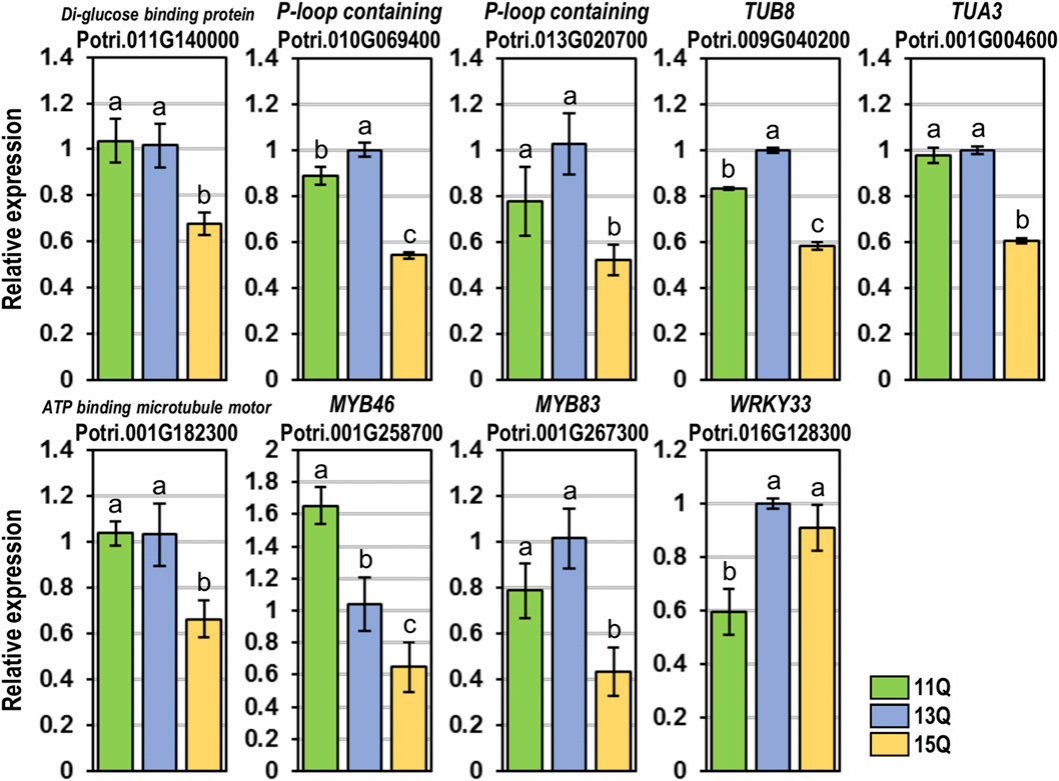
Marker gene expression of *Populus* protoplasts transfected with variant *ANGUSTIFOLIA* alleles. Shown are the averages of three biological replicates ± SE. In each panel, bars labeled with the same letter are not significant different from each other (*P* > 0.05, LSD).

We over-expressed each of the three *PtAN1* alleles in a poplar protoplast system and evaluated the regulatory effect of marker genes utilizing the transient expression assays published previously using the same species (Guo *et al.* 2012). We used the 13Q allele as the comparator since its dual subcellular localization (both cytoplasmic and nuclear localization is consistent with AN homologs reported in other systems including plants and humans (Riefler and Firestein 2001; Minamisawa *et al.* 2011). This analysis revealed that the six microtubule-associated putative targets as well as *MYB46* were significantly upregulated in the 11Q compared to the 15Q variant (Figure 7). Conversely *WRKY33* was significantly upregulated in the 15Q compared to the 11Q variant. These results demonstrated that variation in the length of the polyQ repeat in *PtAN1* which modulated subcellular localization does indeed lead to a significant impact on its transcriptional regulatory function.

## Discussion

Although polyQ repeats have been implicated in changes in protein function across diverse organisms including plants, fungi and humans, the exact mechanism underlying these changes has remained largely elusive. PolyQ repeats have gained considerable attention due to their association and causation of numerous neurodegenerative diseases including Huntington’s disease in humans (Petruska *et al.* 1998; Ross 2002; La Spada and Taylor 2010). As seen in other systems, variation in polyQ repeat length has also been reported in natural populations of plants and animals and shown to be associated with latitudinal variation, response to environmental stresses and reproductive and flowering timing in animals and plants, respectively (Costa *et al.* 1992; Johnsen *et al.* 2007; Liedvogel *et al.* 2009; Caprioli *et al.* 2012). PolyQ repeats, being naturally disordered protein domains, are reported to affect protein folding in a concentration and temperature dependent manner as was illustrated in an *in vitro* assay determining protein dynamics (Deng *et al.* 2012). Additionally, previous reports have shown that heat shock proteins (HSPs) interact with proteins with long polyQ repeats to prevent misfolding and that the availability of HSPs to reduce aggregation of proteins with long polyQ repeats can be impacted by environmental or cellular stresses (Cowan *et al.* 2003; Fujimoto *et al.* 2005).

Despite a lack of consensus on how polyQ repeats modulate protein function, numerous studies have clearly tied expansion and shrinkage of polyQ repeats to transcriptional regulatory efficiency (Gerber *et al.* 1994; Gemayel *et al.* 2015; Kottenhagen *et al.* 2012; Undurraga *et al.* 2012). Moreover, their disproportionate occurrence in eukaryotic transcription factors compared to other functional classes has also been firmly established (Gemayel *et al.* 2015; Willadsen *et al.* 2013; Whan *et al.* 2010). In *Arabidopsis*, PFT1 and ELF3 function were shown to be regulated by polyQ size and both proteins are thought to function as transcription factors. The mechanism by which polyQ size regulates protein activities is still unclear. As such, results presented here offer a possible explanation of how transcriptional regulatory activities are modulated by polyQ repeats. We demonstrated the strong impact of additional two and four glutamine residues on subcellular localization with the 13Q and 15Q alleles exhibiting drastic reduction in nuclear localization.

It is possible that proteins carrying the longer repeat may not fold properly and thus mask their predicted localization motif. Alternatively, the polyQ region may affect potential protein-protein interactions that may be required for nuclear trafficking. The polyQ region in *Pt*AN1 neighbors a putative *RETINOBLASTOMA* binding site and additional acidic polar glutamines may change binding properties of the protein. Given these cumulative observations, it is plausible that this polyQ repeat may alter binding properties necessary for nuclear localization or mask the NLS from being recognized by trafficking proteins.

Curiously, the polyQ repeat in *Pt*AN1 is a novel feature not found in any other homologs of the highly-conserved and copy-number restricted *AN* gene family. Its absence from the *Populus* paralog *Pt*AN2 and the closely related *Salix* genera suggest that this feature arose from a relatively recent evolutionary event which occurred after the Salicoid genome duplication event and subsequent speciation (Tuskan *et al.* 2006). Coevolution network analysis suggested that *PtAN1* and *PtAN2* appear to be on independent evolutionary trajectories, hence the unique occurrence of the expanded polyQ repeat in only one of the otherwise highly homologous loci. On a population level, we identified rare allelic variants differing in the length of the polyQ repeat and occurring across the species range which represents a broad latitudinal gradient. Within the *P. trichocarpa* natural population, we observed a deviation from Hardy-Weinberg equilibrium of allele frequencies among the *PtAN1* variants. Notably, the 15Q variant was found in less than 1% of population samples and, in each case, occurred in heterozygous condition with the 13Q allele. Recalling that we did not observe any geographical patterns related to allelic distribution, it remains to be determined what selection pressures contributed to this deviation in allele frequencies.

Since polyQs have been reported to exhibit mutations rates that are orders of magnitude higher than average single nucleotide polymorphism (Gemayel *et al.* 2015), this novel feature may represent a unique ability to modulate AN function that arose in response to selective pressure uniquely related to *Populus* colonization of its species range over evolutionary time. Further, analysis of polyQ repeats and understanding how they regulate protein function may provide insights into evolution of mechanism to modulate protein function post-speciation.
